# Synthesis of Sulfides and Persulfides Is Not Impeded by Disruption of Three Canonical Enzymes in Sulfur Metabolism

**DOI:** 10.3390/antiox12040868

**Published:** 2023-04-03

**Authors:** Qamarul Hafiz Zainol Abidin, Tomoaki Ida, Masanobu Morita, Tetsuro Matsunaga, Akira Nishimura, Minkyung Jung, Naim Hassan, Tsuyoshi Takata, Isao Ishii, Warren Kruger, Rui Wang, Hozumi Motohashi, Masato Tsutsui, Takaaki Akaike

**Affiliations:** 1 Department of Environmental Medicine and Molecular Toxicology, Tohoku University Graduate School of Medicine, Sendai 980-8575, Japan; 2 Department of Health Chemistry, Showa Pharmaceutical University, Machida, Tokyo 194-8543, Japan; 3 Molecular Therapeutics Program, Fox Chase Cancer Center, Philadelphia, PA 19111-2497, USA; 4 Faculty of Science, York University, Toronto, ON M3J 1P3, Canada; 5 Department of Gene Expression Regulation, Institute of Development, Aging and Cancer, Tohoku University, Sendai 980-8575, Japan; 6 Department of Pharmacology, Graduate School of Medicine, University of the Ryukyus, Okinawa 903-0213, Japan

**Keywords:** cystathionine β-synthase, cystathionine γ-lyase, cysteine persulfide synthase, cysteinyl-tRNA synthetases, 3-mercaptopyruvate sulfurtransferase, reactive persulfides/polysulfides

## Abstract

Reactive sulfur species, or persulfides and polysulfides, such as cysteine hydropersulfide and glutathione persulfide, are endogenously produced in abundance in both prokaryotes and eukaryotes, including mammals. Various forms of reactive persulfides occur in both low-molecular-weight and protein-bound thiols. The chemical properties and great supply of these molecular species suggest a pivotal role for reactive persulfides/polysulfides in different cellular regulatory processes (e.g., energy metabolism and redox signaling). We demonstrated earlier that cysteinyl-tRNA synthetase (CARS) is a new cysteine persulfide synthase (CPERS) and is responsible for the in vivo production of most reactive persulfides (polysulfides). Some researchers continue to suggest that 3-mercaptopyruvate sulfurtransferase (3-MST), cystathionine β-synthase (CBS), and cystathionine γ-lyase (CSE) may also produce hydrogen sulfide and persulfides that may be generated during the transfer of sulfur from 3-mercaptopyruvate to the cysteine residues of 3-MST or direct synthesis from cysteine by CBS/CSE, respectively. We thus used integrated sulfur metabolome analysis, which we recently developed, with 3-MST knockout (KO) mice and CBS/CSE/3-MST triple-KO mice, to elucidate the possible contribution of 3-MST, CBS, and CSE to the production of reactive persulfides in vivo. We therefore quantified various sulfide metabolites in organs derived from these mutant mice and their wild-type littermates via this sulfur metabolome, which clearly revealed no significant difference between mutant mice and wild-type mice in terms of reactive persulfide production. This result indicates that 3-MST, CBS, and CSE are not major sources of endogenous reactive persulfide production; rather, CARS/CPERS is the principal enzyme that is actually involved in and even primarily responsible for the biosynthesis of reactive persulfides and polysulfides in vivo in mammals.

## 1. Introduction

Sulfides and persulfides/polysulfides (RSS_n_H, RSS_n_R, HSS_n_H, CysSS_n_H) are abundant endogenously produced metabolites in cells and tissues of mammals and humans [1,2,3,4]. Because of excess sulfurs on thiol moieties, persulfides possess strong antioxidant properties, which make persulfides superior metabolites compared with thiols. Furthermore, persulfides can manifest dual reactivity properties so that their deprotonated (RSS^–^) and protonated (RSSH) forms act as nucleophiles and electrophiles, respectively [5,6,7,8]. These unique properties make persulfides versatile reactive metabolites in biological signaling mechanisms, which were previously assigned to sulfides or hydrogen sulfide (H_2_S) [9]. Increasing evidence indicates that persulfides play a significant role in cellular regulatory processes and are hypothesized to be as important as other reactive species (e.g., reactive oxygen species and reactive nitrogen species) [7,10,11,12,13].

Persulfides have important roles in various biological phenomena. For example, they were involved in cellular senescence pathways via their reduction/oxidation (redox) modification of 8-nitro-cGMP electrophilic signals [9,14]. Because of strong antioxidant properties, persulfides reportedly acted as excellent scavengers of electrophiles from exogenous sources, such as heavy metals (e.g., methylmercury), and from endogenous sources, such as oxidative/electrophilic stress [5,14,15,16,17,18]. Persulfidation of protein cysteine (CysSH) residues was also an important mechanism in regulating antioxidant responses and reducing processes, such as modification of the Keap1 protein in the Nrf2-Keap1 system and reduction of oxidized proteins, such as those in the thioredoxin (TRX) and peroxiredoxin systems [8,19,20]. Persulfides were recently hypothesized to be involved in electron cycles in the electron transport chain during mitochondrial energy metabolism [1,21,22]. Nevertheless, the primary biosynthesizer of persulfides at the cellular level is still being investigated, with various findings [1,2,7]. Despite these multiple findings, cysteine persulfide (CysSSH) is believed to be pivotal in the formation of persulfides/polysulfides.

The canonical pathway of sulfide and CysSSH production involves two CysSH synthesis/transsulfuration pathway enzymes—cystathionine β-synthase (CBS) and cystathionine γ-lyase (CSE)—and one CysSH metabolism pathway enzyme: 3-mercaptopyruvate sulfurtransferase (3-MST) [2,23,24]. Previously in our laboratory, we found that cysteinyl-tRNA synthetases (CARSs) involved in the synthesis of persulfides and mitochondrial CARS (CARS2) were responsible for the activity of cysteine persulfide synthases (CPERS) in producing CysSSH in mitochondria [1]. With regard to persulfide levels, we also discovered that HEK293T cells that had their expression of CBS and CSE silenced showed no significant reduction in CysSSH metabolites, whereas CysSSH reduction was substantial in the CARS2 knockout (KO) cell line [1]. We also determined that persulfide synthesis via the CBS/CSE pathway was inefficient because of the high *K_m_* value of the reaction under normal physiological conditions [1,2]. Our previous results confirmed a significant reduction in persulfide synthesis in CARS2-KO cells but not in CBS and CSE knockdown cells, so these findings raise doubts about the importance of canonical sulfide-producing enzymes, such as CBS, CSE, and to some extent 3-MST, during persulfide synthesis. To assess the contribution of CBS, CSE, and 3-MST in persulfide production compared with the importance of CARS2, we generated triple-KO mice for CBS, CSE, and 3-MST and CARS2-deficient heterozygous mice (*Cars2^+/−^*), and we quantitatively analyzed persulfide levels in various tissues of these KO and CARS2-deficient mice.

## 2. Materials and Methods

### 2.1. Materials

β-(4-Hydroxyphenyl)ethyl iodoacetamide (HPE-IAM) was obtained from Chem-Impex (Wood Dale, IL, USA). Monobromobimane (Br-bimane) was obtained from Merck (Darmstadt, Germany). Liquid chromatography chemicals were obtained from FUJIFILM Wako Pure Chemical (Osaka, Japan). A Taq polymerase kit was obtained from Takara Bio (Shiga, Japan). A sequencing kit was obtained from Thermo Fisher Scientific (Waltham, MA, USA). For Western blotting, we used the ECL Prime Western Blotting Detection Reagent from GE Healthcare (Chicago, IL, USA), as well as antibodies such as anti-GAPDH (sc-25778) and anti-3-MST (sc-376168) obtained from Santa Cruz Biotechnology (Santa Cruz, CA, USA); anti-CBS (3E1) obtained from Abnova (Taipei, Taiwan); anti-CARS2 provided by Prof. Hideshi Ihara (Osaka Prefecture University); and anti-CSE provided by Prof. Yoshito Kumagai (University of Tsukuba).

### 2.2. Animals

**Figure 1:**
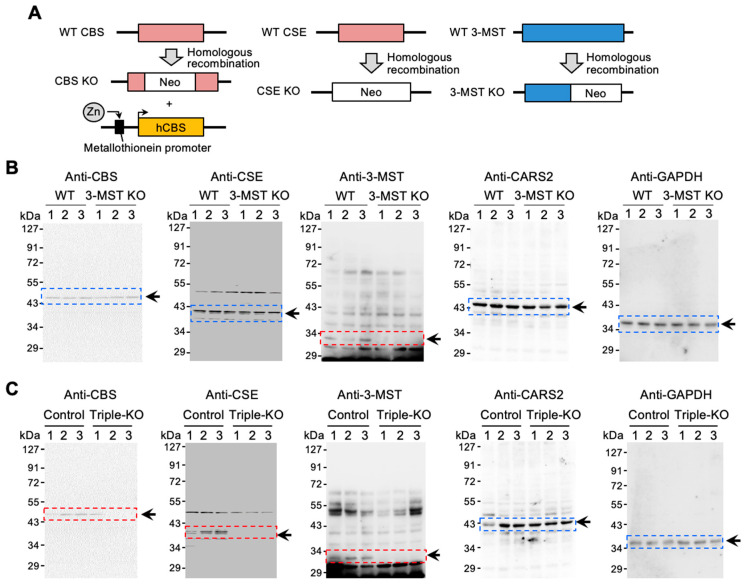
Generation of CBS/CSE/3-MST triple-KO mice. (**A**) CBS/CSE/3-MST triple-KO mice were generated by cross-breeding with single-KO CBS, CSE, and 3-MST mice. The triple-KO mice contained Tg-hCBS to prevent lethality in CBS KO mice. hCBS cDNA was conjugated to the metallothionein promoter controlled by zinc. Control mice had only Tg-hCBS along with wild-type (WT) CBS, CSE, and 3-MST. (**B**) Western blotting of CBS, CSE, 3-MST, and CARS2, obtained from the brains of control and 3-MST KO mice. (**C**) Western blotting of CBS, CSE, 3-MST, and CARS2, obtained from the brains of control and triple-KO mice. GAPDH was used as the internal control. The arrows and boxes outlined with dashed lines indicate the primary bands at the expected molecular weights.

All experimental procedures were conducted according to the Regulations for Animal Experiments and Related Activities at Tohoku University, reviewed by the Institutional Laboratory Animal Care and Use Committee of Tohoku University, and approved by the President of Tohoku University. For the generation of 3-MST KO mice, we inserted a gene trap vector into the first intron of the mouse *3-MST* gene in mouse embryonic stem cell lines and generated chimera mice according to the standard protocol [25]. Single-heterozygous CBS mice (*Cbs^+/−^*) [26], CSE mice (*Cth^+/−^*) [27], and 3-MST mice (*Mpst^+/−^*) were mated with each other to produce triple-heterozygous CBS/CSE/3-MST mice. The triple-heterozygous mice were then intercrossed, and CBS/CSE/3-MST triple-KO mice were generated (Figure 1A). Due to the lethality in KO CBS mice, triple-heterozygous KO mice were crossed with human CBS transgenic (Tg-hCBS) mice before the generation of triple-KO mice to avoid lethality in immature mice. The expression of human CBS in Tg-hCBS mice can be activated by supplying zinc water. Therefore, to ensure survivability of the mice to adulthood, zinc water was supplied and then stopped 2 weeks before the experiment [28]. Single CSE KO mice used in Figure S2 are a different strain from the CSE KO mice used to create the triple KO mice [29].

CARS2-deficient heterozygous mice (*Cars2^+/−^*) were from previously generated CARS2-deficient mice [1]. The genetic modifications of the mice were confirmed by using polymerase chain reaction (PCR) and direct sequencing. Briefly, genomic samples from mice were amplified by using the Taq polymerase PCR kit, with the PCR reaction as follows: initial denaturation at 94 °C for 1 min, followed by 95 °C for 30 s, 60 °C for 30 s, and 72 °C for 30 s for 35 cycles. Primers (0.4 μM) specific for the target genes were used. Resultant PCR fragments were processed via gel electrophoresis in 2% agar for confirmation and compared against known DNA size markers. Table S1 provides all primers used for the different genotypes.

### 2.3. Knockdown of CBS and CSE

For knockdown of CBS and CSE expression, we used small interfering RNAs: CBS, CBSHSS101428 (Invitrogen, Waltham, MA, USA), and CSE, CTHHSS102447 (Invitrogen, Waltham, MA, USA). We performed small interfering RNA transfection using Lipofectamine RNAiMAX (Invitrogen, Waltham, MA, USA) according to the manufacturer’s instructions.

### 2.4. Measurement of Persulfides and Derivative Metabolites by Means of Br-Bimane Labeling

Liquid chromatography-electrospray ionization–tandem mass spectrometry (LC-ESI–MS/MS) combined with Br-bimane trapping was used to measure sulfur metabolites produced, according to our previous studies [2]. Metabolites from mouse tissue were extracted using a 5 mM Br-bimane with 100% methanol and then homogenized. Lysates were harvested and incubated at 37 °C for 15 min. After centrifugation, aliquots of the supernatants were diluted 10–100 times with distilled water containing known amounts of isotope-labeled internal standards. We used an Agilent 6430 Triple Quadrupole LC/MS (Agilent Technologies, Santa Clara, CA, USA) to perform LC-electrospray ionization–MS/MS. The ionization was achieved by using electrospray in the positive mode, and polysulfide derivatives were identified and quantified by means of multiple reaction monitoring. The MRM parameters and HPLC conditions for LC–MS/MS analysis using Br-bimane followed our report [2].

### 2.5. Measurement of Persulfides and Derivative Metabolites by Means of HPE-IAM Labeling

LC-ESI–MS/MS combined with HPE-IAM trapping was used to measure sulfur metabolites produced, according to our previous studies [1,30,31,32]. Metabolites were extracted using a solution containing 5 mM HPE-IAM with 70% methanol and then homogenized. Lysates were harvested and incubated at 37 °C for 20 min and then centrifuged at 15,000× *g*. After centrifugation, aliquots of the lysate supernatants were diluted 20–50 times with 0.1% formic acid containing known amounts of isotope-labeled internal standard (50–200 nM). Sulfide metabolites in each sample were quantified using the Nexera UHPLC system (Shimadzu, Kyoto, Japan) and LCMS-8060 (Shimadzu, Kyoto, Japan) LC-ESI–MS/MS. Samples were injected and separated by means of the YMC-Triart C18 column (50 × 2.0 mm inner diameter), eluted with a methanol mobile phase through a linear gradient (0–90%) for 15 min in the presence of 0.1% formic acid at a flow rate of 0.2 mL/min at 40 °C. CysSH, CysSSH, glutathione (GSH), glutathione persulfide (GSSH), glutathione disulfide (GSSG), homocysteine (homoCysSH), homocysteine persulfide (homoCysSSH), cystine, hydrogen sulfide anion (HS^–^), hydrogen disulfide anion (HSS^–^), and thiosulfate (HS_2_O_3_^–^) were identified and quantified by multiple reaction monitoring (MRM) on the basis of their specific parameters (Table S2), as previously performed [1,2,31].

### 2.6. Statistical Analysis

Data are means  ±  s.d. of at least three independent experiments unless otherwise specified. We analyzed comparisons among multiple groups of mice or cell lines with a one-way ANOVA with Tukey’s test, whereas we used Student’s *t*-test for comparisons of continuous variables. We set *p*-values of less than 0.05 as significant. We used GraphPad Prism 9 (GraphPad Software) for statistical analysis.

## 3. Results

### 3.1. 3-MST Mice Demonstrated No Reduced CysSSH and GSSH

**Figure 2:**
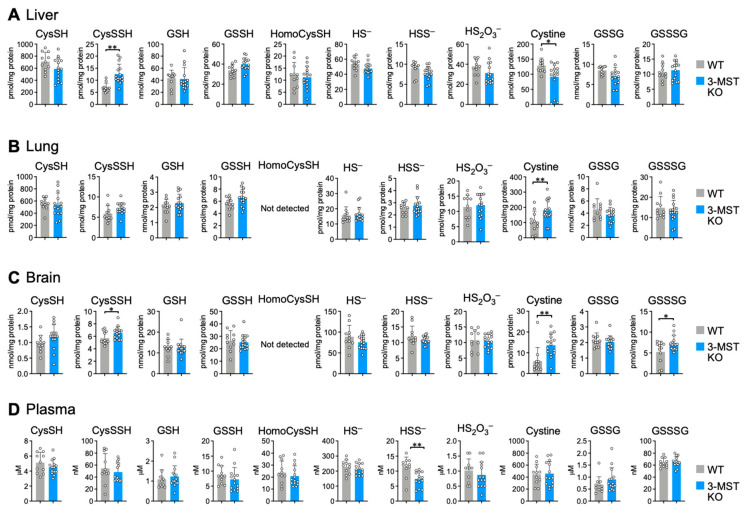
Sulfur metabolome analysis in 3-MST KO mice. Endogenous production of CysSSH, GSSH, and related compounds was identified by means of HPE-IAM labeling with LC–MS/MS analysis of the liver (**A**), lung (**B**), brain (**C**), and plasma (**D**) obtained from 8- to 10-week-old WT and 3-MST KO mice. Data are means ± s.d. *n* = 11 (WT) and 15 (3-MST KO). * *p* < 0.05; ** *p* < 0.01, as determined by Student’s *t*-test.

To determine the importance of canonical enzymes in persulfide synthesis, we used sulfur metabolome analysis to study 3-MST KO mice. We first generated 3-MST KO mice by inserting a gene trap vector into an intron in mouse 3-MST; we confirmed the absence of 3-MST protein expression using Western blotting (Figure 1A,B). The expression of CBS, CSE, and CARS2 was unchanged in 3-MST KO mice compared with wild-type mice. (Figure 1B). We therefore quantified CysSSH, GSSH, and other persulfides and their derivatives in liver, lung, and brain tissues and plasma from 3-MST KO mice using LC–MS/MS. We found no significant differences in the synthesis of CysSSH, GSSH, and other persulfides in all the samples that we examined (Figure 2). Although very few results showed some discrepancies, such as the increased level of CysSSH in the liver and the decreased level of HSS^–^ in the plasma, the outcome of this experiment demonstrated that the synthesis of persulfides, including CysSSH, GSSH, and persulfide derivatives, does not depend on 3-MST activity. Section 4 provides a plausible explanation of these discrepancies.

### 3.2. Triple-KO Mice Showed No Reduced CysSSH and GSSH

To ascertain the importance of canonical enzymes in persulfide synthesis, single-KO CBS, CSE, and 3-MST mice were crossed to generate triple-heterozygous mice. These triple-heterozygous mice were then crossed to obtain triple-KO mice. CBS KO mice die within 5 weeks after birth [26]; therefore, Tg-hCBS mice containing a zinc-inducible metallothionein promoter that is activated by zinc were crossed with triple-heterozygous mice before the generation of triple-KO mice to avoid lethality in immature mice (Figure 1A) [28]. To ensure comparable results, both control mice and triple-KO mice had the Tg-hCBS expression cassette inserted, and CBS expression was controlled by supplying water containing zinc (Figure 1A). As Figure 1C shows, tissues of triple-KO mice manifested no expression of CBS, CSE, and 3-MST proteins when water without zinc was provided for 2 weeks.

**Figure 3:**
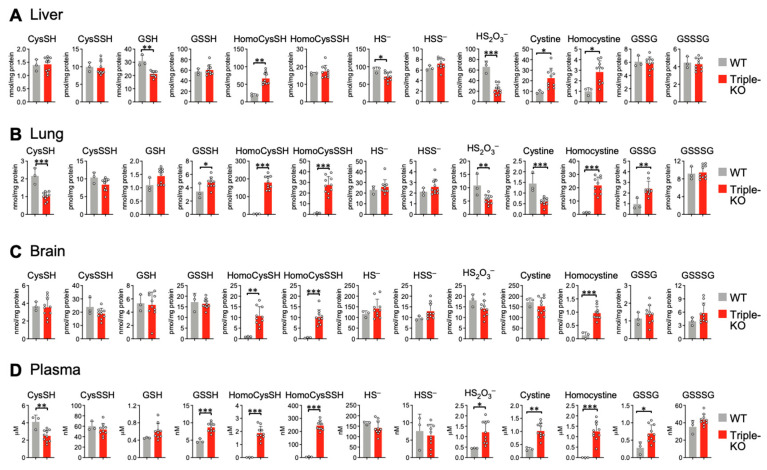
Sulfur metabolome analysis in triple-KO mice. Endogenous production of CysSSH, GSSH, and related compounds was identified by means of HPE-IAM labeling with LC–MS/MS analysis of the liver (**A**), lung (**B**), brain (**C**), and plasma (**D**) obtained from control and triple-KO mice. Mice were males, 14–16 weeks old. Data are means ± s.d. *n* = 3 (WT) and 10 (triple-KO). * *p* < 0.05; ** *p* < 0.01; *** *p* < 0.001, as determined by Student’s *t*-test.

To investigate persulfide synthesis in triple-KO mice, we used LC–MS/MS analysis to measure CysSSH and GSSH in various tissues from triple-KO and control mice. We also measured persulfides and their derivatives in plasma, the presence of which is an indicator of global physiology metabolites. Triple-KO mice demonstrated no significant differences when compared with control mice in terms of CysSSH synthesis in liver, lung, brain, and plasma tissues (Figure 3). The GSH level significantly decreased in livers of triple-KO mice (Figure 3A) but remained similar in lungs (Figure 3B), brains (Figure 3C), and plasma (Figure 3D) from both control and triple-KO mice. The GSSH level significantly increased in lungs (Figure 3B) and plasma (Figure 3D) of triple-KO mice compared with control mice, which were similar in liver (Figure 3A) and brain (Figure 3C) tissues. The discrepancies between these tissue GSSH metabolite levels may be due to various reasons, such as oxidative stress conditions caused by the generation of triple-KO mice and different gene expression levels of sulfide-metabolizing enzymes in the specific tissues; Section 4 addresses these issues. Nevertheless, our findings indicated that deletion of CBS, CSE, and 3-MST genes in mice did not affect persulfide synthesis. These results indicate that CARS2 functionally complements the three enzymes in the triple-KO mice.

### 3.3. Triple-KO Mice Manifested Impaired CysSH Production and Aberrant Sulfide Metabolism

To determine the effects of KO genes in triple-KO mice, we also measured metabolites that are involved in the metabolic pathways for these genes. Since CBS and CSE are a part of the CysSH metabolic pathway [2,33,34], we measured CysSH using LC–MS/MS analysis. We found significantly reduced CysSH metabolite levels in lung tissues (Figure 3B) and plasma (Figure 3D) of triple-KO mice. We expected this finding because disruption of CBS and CSE enzymes reportedly prevented CysSH synthesis (Figure S2) [1,2]. To continue our investigation, we measured homoCysSH, which is an intermediate metabolite in the CysSH synthesis pathway via CBS and CSE, by means of LC–MS/MS analysis in triple-KO mice. We detected significantly elevated levels of homoCysSH in triple-KO mice. We found significantly increased homoCysSSH levels in lung (Figure 3B) and brain (Figure 3C) tissues, as well as in plasma, but not in liver (Figure 3A) tissues of triple-KO mice. Accumulations of homoCysSH may increase the chance of polysulfidation of homoCysSH, which would result in the formation of homoCysSSH [2,5,7,15,35]. Thus, our results for CysSH, homoCysSH, and homoCysSSH were related to the effects of CBS and CSE KO in triple-KO mice.

With regard to 3-MST in triple-KO mice, we utilized LC–MS/MS analysis to measure sulfide metabolites and their derivatives (HS^–^, HSS^–^, and thiosulfate). Levels of thiosulfate significantly decreased in liver and lung tissues (Figure 3A,B) but significantly increased in plasma (Figure 3D). The decrease in thiosulfate in lung tissues may be explained by a possible chemical reaction for thiosulfate to transfer its sulfur residue to homoCysSH and GSH to form their persulfide derivatives. This mechanism, in turn, may increase the production of homoCysSSH and GSSH in both lung tissues and plasma (Figure 3B,D). However, the thiosulfate level of brain and plasma was similar to or significantly higher than that in each control tissue, respectively, which indicates that sulfurtransferase activity was sustained by some potent sulfide biosynthesis pathways occurring in vivo even though all three genes were deleted. While it is possible that thiosulfate is derived via oxidative degradation of various hydropersulfides/polysulfides, a rhodanese family enzyme, named thiosulfate sulfurtransferase (TST), may possess sulfur transfer activity to generate thiosulfate similar to that of other canonical enzymes. Therefore, the varied levels of thiosulfate in our triple-KO mice may be because of compensatory activity of TST, as described in the Section 4.

### 3.4. CysSSH Synthesis Was Disrupted in Cars2^+/−^ Mice

**Figure 4:**
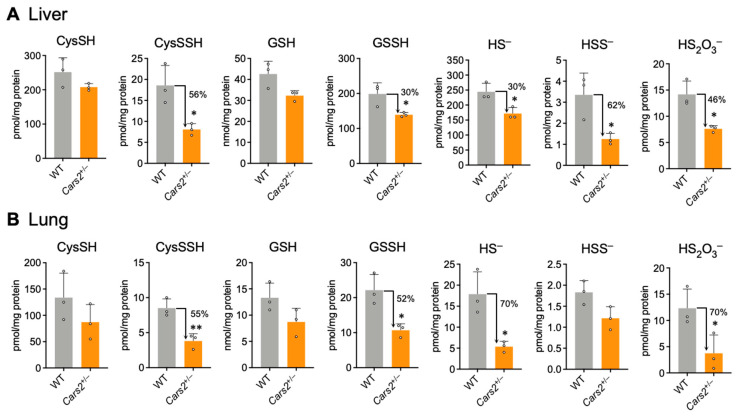
Sulfur metabolome analysis in *Cars2*^+/−^ mice. Endogenous levels of sulfur metabolites were identified as those of HPE-IAM adducts by using LC–MS/MS analysis of liver (**A**) and lung (**B**) tissues of 10- to 16-week-old WT and *Cars2*^+/−^ mice. Data are means + s.d. *n* = 3. * *p* < 0.05; ** *p* < 0.01, determined by Student’s *t*-test.

To clarify the importance of canonical enzymes in the persulfide synthesis pathway, we investigated another known persulfide synthesis pathway. We previously identified CARSs as serving as the principal CysSSH synthases in vivo [1]. Here, we used CARS2-deficient mice to highlight the importance of CARS2 in persulfide synthesis. Since deletion of the *Cars2* gene in mice proved to be embryonic lethal [1], we used previously generated *Cars2* heterozygous mice (*Cars2^+/^*^–^) in this study. *Cars2^+/–^* mice had a 1-bp insertion in exon 3 of the *Cars2* gene, which led to impaired CARS2 protein and persulfide synthesis in *Cars2^+/−^* mice. Western blot assay results using tissues from *Cars2^+/-^* mice showed no change in protein expression level of 3-MST, CBS, and CSE between wild-type and Cars2-deficient mice [1]. This result indicates that there is no complementary increase in expression of the three canonical enzymes, at least under regular laboratory housing conditions. Since CARS2 protein uses CysSH as a substrate to synthesize CysSSH [1], and CysSH is subsequently utilized for the synthesis of other persulfides, we used LC–MS/MS analysis to measure CysSH and CysSSH in liver and lung tissues of *Cars2^+/−^* mice. Levels of CysSH were not significantly different in both wild-type (WT) and *Cars2^+/−^* mice, but CysSSH synthesis was significantly reduced to 56% and 55% in liver and lung tissues, respectively, in *Cars2^+/−^* mice (Figure 4). This finding was similar to results found in our previous experiment, in which the in vitro model using CARS2-KO HEK293T cells showed reduced CysSSH synthesis but no effects on the synthesis of CysSH [1] (Figure S1). Thus, reduced CysSSH synthesis here in *Cars2^+/−^* mice highlighted the CARS2 enzyme as the most important physiological CPERS.

### 3.5. Persulfides and Their Derivatives Are Reduced in Cars2^+/−^ Mice

We also used LC–MS/MS analysis to measure the production of persulfides such as GSSH and various sulfide metabolites, including HS^–^, HSS^–^, and thiosulfate in *Cars2^+/−^* mice. Measurement showed that levels of CysSSH, GSSH, and other derivative compounds were significantly reduced in the liver and lung tissues of *Cars2^+/−^* mice compared with those of WT mice. We observed reductions of 56% and 55% of CysSSH, and 30% and 52% of GSSH, respectively, in the liver and lung tissues of *Cars2^+/−^* mice, as well as reductions in other sulfide derivatives (Figure 4). However, levels of CysSH and GSH, precursors for the synthesis of each persulfide, showed no significant differences in *Cars2^+/−^* mice compared with WT mice (Figure 4A,B). These results indicate that the synthesis of persulfides, such as CysSSH, GSSH, and the sulfide derivatives (HS^–^, HSS^–^, and thiosulfate), depends on CARS2 in mouse tissues.

The present study shows that the canonical sulfide-producing enzymes—CBS, CSE, and 3-MST—are not essential for the synthesis of both sulfides and persulfides. Although CBS and CSE produce CysSSH by using cystine as substrate, our current study demonstrates that cysteinyl-tRNA synthetase (CARS) plays a major role in the synthesis of CysSSH in vivo.

## 4. Discussion

**Figure 5:**
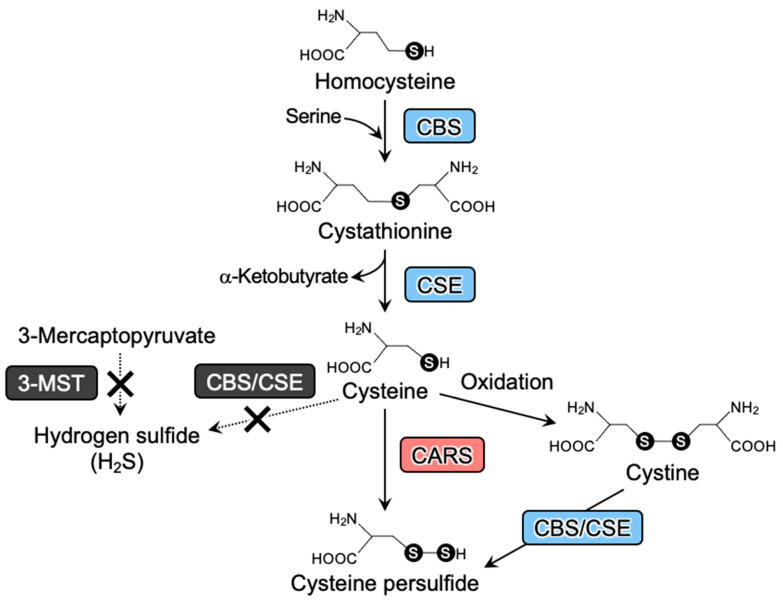
Canonical and true pathways for persulfide biosynthesis. The canonical pathway for persulfide production is composed of sulfurtransferase enzymes (e.g., CBS and CSE; bule boxes). CARS mediates the true and major pathway that mediates persulfide biosynthesis (red box). 3-MST, CBS/CSE may not be involved solely in the sulfide production (black box).

Our present study demonstrates that the absence of canonical H_2_S-producing enzymes—CBS, CSE, and 3-MST—does not affect either sulfide or persulfide synthesis (Figure 5). To the best of our knowledge, this report presents the first in vivo study of the role of canonical H_2_S-producing enzymes (CBS, CSE, and 3-MST) in the biosynthesis of persulfides using a mouse model in which all three genes were deleted. Until now, endogenous persulfides were thought to be produced via H_2_S oxidation resulting from CBS, CSE, and 3-MST [2,24] enzymatic activities. In contrast to this earlier interpretation, significant reductions in persulfide metabolites were not observed for the triple-KO mice in this study. We previously showed, however, a significant decrease in CysSSH in CARS2-deficient mice [1], which we consistently observed in our current study. This finding thus supports CARS2 as the primary CPERS expressed in vivo.

However, another finding that we obtained from our study is that CBS, CSE, and 3-MST regulated levels of metabolites related to persulfide metabolism in mouse tissues. In particular, CysSH, homoCysSH, and homoCysSSH levels were heavily affected by disruptions in CBS and CSE. CBS and CSE enzymes are involved in the synthesis of CysSH, with homoCysSH being the intermediate metabolite in the pathway of CysSH synthesis from methionine. Deletion of CBS and CSE genes increased homoCysSH and reduced CysSH levels in vivo, which was confirmed in our previous study with HEK293T cells [1] and in reports by others [26,28]. Although CBS and CSE may contribute to the synthesis of H_2_S under physiological conditions, their substantial contributions depend on the activity levels of this enzyme expressed in different cells and tissues as well as under various cellular metabolic conditions [1,2,9,36,37,38,39]. For example, these canonical enzymes and Cars2 are known to be highly expressed in cancer tissues such as human Basal-like breast cancers and colorectal cancer tissues [40,41]. In the breast cancer tissues where CBS and CSE are highly expressed, CBS and CSE play important roles in sulfur metabolism and tumorigenesis [40]. Additionally, H_2_S production by CSE reportedly increased under the oxidative conditions of hyperhomocysteinemia [36,38,39]. We found that, with the increased homoCysSH conditions in triple-KO mice, significantly higher accumulations of homocystine and homoCysSSH occurred in the tissues of triple-KO mice than those of WT mice (Figure 3). One plausible explanation for the homoCysSSH increase is the possible involvement of putative persulfide synthases yet to be identified, which may use homoCysSH as a substrate to generate homoCysSSH. In any case, our triple-KO mice study, showing increased homoCysSSH in lung and brain tissues, indicates that CBS and CSE are, at the very least, not the primary regulators of endogenous persulfide production in vivo.

The accumulation of homoCysSH and homoCysSSH in triple-KO mice caused aberrant sulfur metabolism, which may explain the differences in the amounts of GSH, GSSH, and thiosulfate (HS_2_O_3_^–^) in various tissues of these mice. In addition, the amino acid sequence of 3-MST had high homology with that of TST, with a conserved sulfurtransferase protein activity domain [42]. The gene structures of 3-MST and TST are close to each other, within 2 kb, so a bidirectional gene pair suggests that 3-MST and TST share a common enhancer/promoter region. Also, increased TST expression was noted in 3-MST KO mice [23]. This finding may indicate that both 3-MST and TST work in complementary ways in sulfur metabolism, as was observed for 3-MST KO mice for the triple-KO condition that we used here. In a recent report, it has been revealed that the 3-MST protein functions as a protein persulfidase, with the cytoplasmic form of CARS, known as CARS1, also expected to primarily operate in a similar capacity. Considering the potential for these canonical enzymes to collaborate as protein persulfidases [43]. Further research should be conducted to explore this possibility. Thus, studying the relationship between 3-MST, TST, and CARS in terms of sulfide metabolism and protein persulfidase would be interesting for future investigations.

CysSSH was previously reported to be formed in an active center of a cysteine residue as an intermediate species during a sulfur transfer reaction catalyzed by 3-MST [24]. According to this potential catalytic scheme, CysSSH formed in 3-MST may then react with GSH to produce GSSH. In contrast, other studies [1,2,35] suggested that the synthesis of CysSSH via CBS and CSE mostly occurs under pathophysiological conditions such as oxidative stress. However, we found here that persulfide biosynthesis, at least as observed in triple-KO mice, is not likely to depend on CBS, CSE, and 3-MST (Figure 5).

The discrepancy between results of our study and those of others may be due to the different techniques used to measure sulfide and persulfides. For example, monobromobimane (MBB) and *N*-ethylmaleimide (NEM) are commonly utilized as polysulfide probes during high performance LC and LC–MS/MS measurement of persulfides [1,31]. MBB and NEM are harsh electrophiles that may participate in shifting the hydrolysis equilibrium by a nucleophilic attack on a sulfur residue of polysulfides, which would lead to polysulfide decomposition and result in artefactual sulfide determinations [1,6,30,31,32]. Such a technical flaw may be prevented by means of the polysulfide-stabilizing activity conferred by *N*-iodoacetyl l-tyrosine methyl ester (TME-IAM) and HPE-IAM [30,31,32]. In our study, therefore, we used HPE-IAM as a highly reliable reagent for sulfide/persulfide measurement via LC–MS/MS-based sulfur metabolome analysis, which allowed us to perform extremely accurate, quantitative, and reproducible measurements, as compared with the much less precise and classical approach via MBB and NEM derivatization.

## 5. Conclusions

Our current study exploited the novel and elegantly integrated animal model of CBS/CSE/3-MST triple-KO mice, which was, in fact, an efficient tool for investigating the sulfur metabolome, and it demonstrated that all three genes are not vital for CysSSH biosynthesis in vivo under physiological conditions. We also confirmed the primary role of CBS and CSE genes in the CysSH biosynthesis pathway rather than persulfide production. This study may also clarify the predominant role of CARS2/CPERS in the biosynthesis of persulfides and in sulfur metabolism as opposed to the role of three other canonical sulfide/persulfide-generating enzymes—CBS/CSE/3-MST. Moreover, our study may suggest a new study area of sulfur-based redox biology and medicine, in which reactive sulfur species and persulfides are potential therapeutic targets in various diseases that are associated with impaired endogenous persulfide production.

## Data Availability

The data are contained within the manuscript.

## Supplementary Materials

The following supporting information can be downloaded at: https://www.mdpi.com/article/10.3390/antiox12040868/s1, Figure S1: Effect of CBS and CSE knockdown on CysSH, GSH, and related sulfide/persulfide metabolites formed in HEK293T cells with or without CARS2 expression; Figure S2: Sulfur metabolome analysis in CSE KO mice; Table S1: Primers used for different genotypes of KO mice; Table S2: MRM parameters used for LC-ESI-MS/MS analyses.
