# Supplementary material for: Synthesis of Sulfides and Persulfides Is Not Impeded by Disruption of Three Canonical Enzymes in Sulfur Metabolism
Source: Antioxidants (Basel). 2023 Apr 3;12(4):868. doi: 10.3390/antiox12040868 (PMC10135671; doi:10.3390/antiox12040868)
Supplement: Supplementary file 1 [file antioxidants-12-00868-s001.zip › antioxidants-2221423-supplementary.pdf]

Supplementary Materials

Synthesis of sulfides and persulfides is not impeded by disruption of three canonical enzymes in sulfur metabolism

Qamarul Hafiz Zainol Abidin[1,†], Tomoaki Ida[1,†], Masanobu Morita[1,†,*], Tetsuro Matsunaga[1,†], Akira Nishimura[1], Minkyung Jung[1], Naim Hassan[1], Tsuyoshi Takata[1], Isao Ishii[2], Warren Kruger[3], Rui Wang[4], Hozumi Motohashi[5], Masato Tsutsui[6,*], Takaaki Akaike[1,*]

[†]These authors contributed equally to this work.

*Corresponding authors: morita@med.tohoku.ac.jp (M. Morita);

tsutsui@med.u-ryukyu.ac.jp (M. Tsutsui);

takaike@med.tohoku.ac.jp (T. Akaike)

This PDF file includes:

- Supplementary Figure (**Figure S1 and Figure S2**)
- Supplementary Tables (**Table S1 and Table S2**)

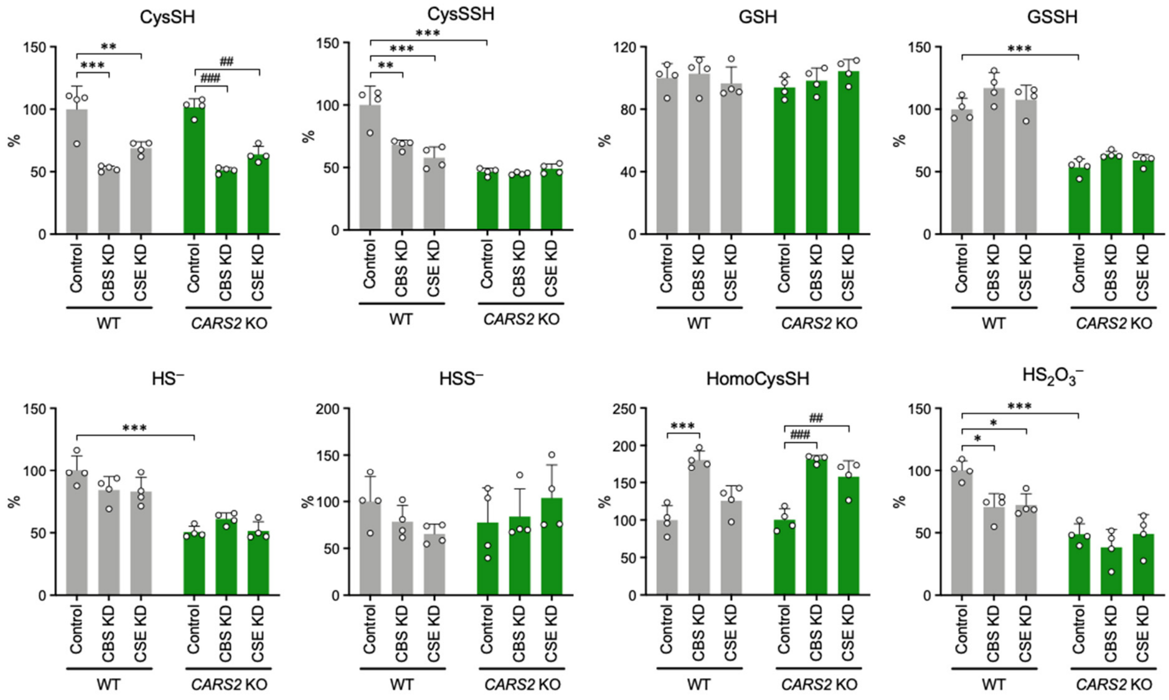

Figure S1. Effect of CBS and CSE knockdown on CysSH, GSH, and related sulfide/persulfide metabolites formed in HEK293T cells with or without CARS2 expression. Knockdown of CBS and CSE was performed using the following small interfering RNAs: CBS, CBSHSS101428 (Invitrogen), and CSE, CTHHSS102447 (Invitrogen). Intracellular levels of CysSSH, GSSH, and related sulfide derivatives in WT and CARS2 KO cells with CBS or CSE knocked down (KD). Data are means ± s.d. $n = 3$. *$p < 0.05$ (vs. WT mock); **$p < 0.01$ (vs. WT mock); ***$p < 0.001$ (vs. WT mock); $^{\#\#}p < 0.01$ (vs. CARS2 KO mock); $^{\#\#\#}p < 0.001$ (vs. CARS2 KO mock), as determined by Student's t-test.

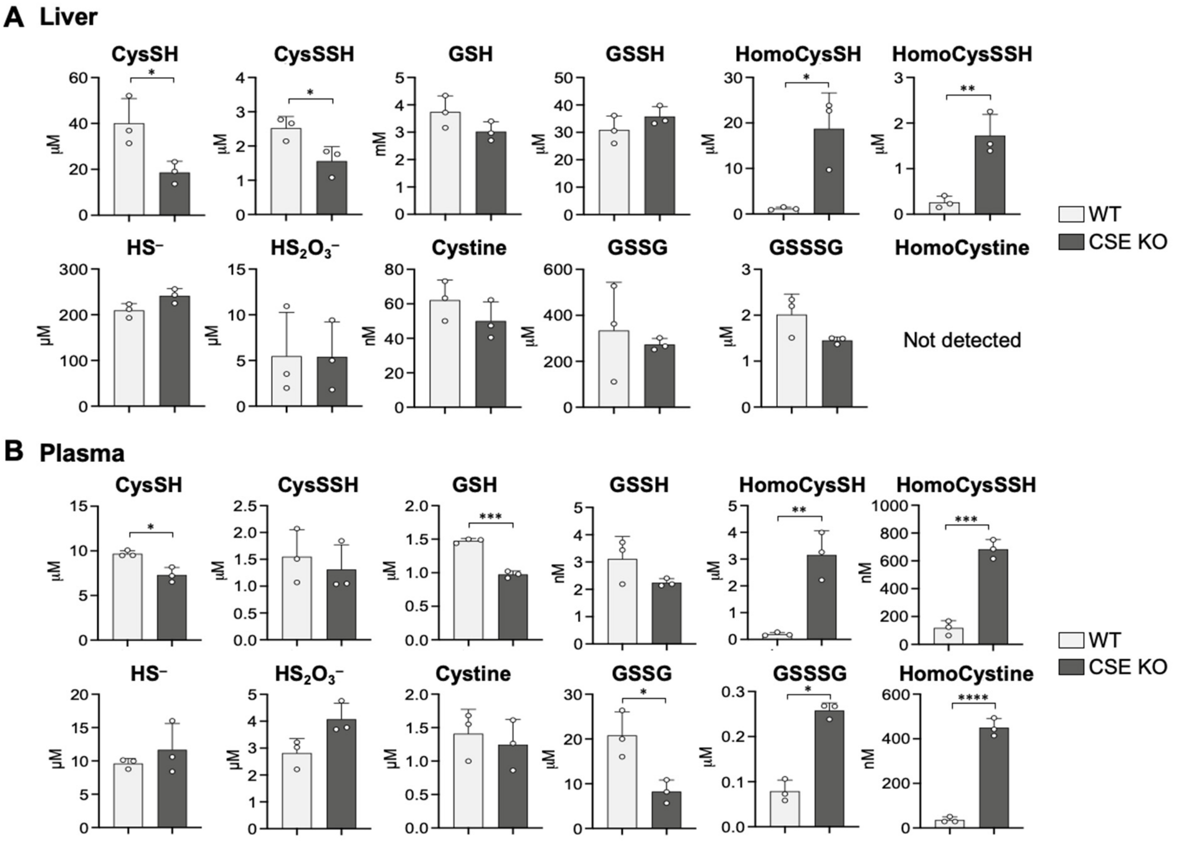

Figure S2. Sulfur metabolome analysis in CSE KO mice.

Endogenous production of CysSSH, GSSH, and related compounds was identified by means of Br-bimane labeling with LC–MS/MS analysis of plasma obtained from 9- to 10-week-old WT and CSE KO mice. In the measurement method using HPE-IPM, sulfur metabolites were measured based on the protein amount, while in the measurement method using Br-bimane labeling, the tissue weights were converted to volume, and the sulfur metabolites per unit volume were measured. Data are means ± s.d. $n = 3$. $*p < 0.05$; $**p < 0.01$, $***p < 0.005$; $****p < 0.0001$, as determined by Student's t-test.

Table S1. Primers used for different genotypes of KO mice.

Primer names	Primer sequences for genotype	Genotype for gene
CARS2 forward	5’-GTGCGAGAAGCCAGAAAA-3’	*Cars2*
CARS2 reverse	3’-AAGGGTCACAAGTACTAGGA-5’	
CBS forward	5’-ACTACGATGACACCGCCGAG-3’	*Cbs*
CBS reverse	3’-GAGTAGCGTGTCCTTCCTGA-5’	
Tg-hCBS forward	5’-CGAAGGGGAGCCTGGAGAAG-3’	Human CBS Transgenic
Tg-hCBS reverse	3’- CTGGCAAGATTTTTGGAGATTTTG-5’	
CSE forward	5’-TGCCGACCAATAAGCAGGGC-3’	*Cth*
CSE reverse 1	3’-TGAAGGGCCCGGTCAGGAGCC-5’	
CSE reverse 2	3’-ACTAAAAGCAACGGCCAGACC-5’	
3-MST forward	5’-GCTCCTCACAGCCGCTGAAG-3’	*Mpst*
3-MST reverse	3’-CAGGTTCTGATTCAGCCAGTG-5’	

Table S2. MRM parameters used for LC-ESI–MS/MS analyses.

Compound	Polarity	Precursor ion (*m/z*)	Product ion (*m/z*)	Collision energy (V)
CysS-HPE-AM	+	298.9	121.0	-29
[$^{13}C_1$] CysS-HPE-AM	+	299.9	121.0	-29
CysS-S-HPE-AM	+	330.8	121.0	-29
CysS-[$^{34}S_1$]-HPE-AM	+	332.8	121.0	-29
GS-HPE-AM	+	484.9	356.3	-18
[$^{13}C_2$, $^{15}N_1$]GS-HPE-AM	+	488.1	359.3	-18
GS-S-HPE-AM	+	516.9	388.3	-18
GS-[$^{34}S_1$]-HPE-AM	+	518.9	121.0	-19
HomoCysS-HPE-AM	+	312.9	121.0	-32
d4-HomoCysS-HPE-AM	+	316.9	121.0	-32
HomoCysSS-HPE-AM	+	344.8	121.0	-28
d4-HomoCysSS-HPE-AM	+	348.8	121.0	-28
Bis-S-HPE-AM	+	388.9	121.0	-30
Bis-[$^{34}S_1$]-HPE-AM	+	390.9	121.0	-30
Bis-SS-HPE-AM	+	420.9	121.0	-23
Bis-[$^{34}S_2$]-HPE-AM	+	424.9	121.0	-23
HS_2O_3-HPE-AM	–	290.0	208.2	14
H[$^{34}S_2$]O_3-HPE-AM	–	294.0	210.2	14
Cystine	+	241.0	152.0	-14
[$^{13}C_2$]Cystine	+	243.0	153.0	-14
GSSG	-	611.2	306.2	23
[$^{13}C_4$,$^{15}N_2$]GSSG	-	617.2	309.2	23
GSSSG	-	643.2	272.4	26
GS-[$^{34}S_1$]-SG	-	645.2	272.4	26
Homocystine	+	269.0	136.0	-12
d8-Homocystine	+	277.0	140.0	-12
